# Barriers and facilitators to HPV vaccination in primary care practices: a mixed methods study using the Consolidated Framework for Implementation Research

**DOI:** 10.1186/s12875-018-0750-5

**Published:** 2018-05-07

**Authors:** Jane M. Garbutt, Sherry Dodd, Emily Walling, Amanda A. Lee, Katharine Kulka, Rebecca Lobb

**Affiliations:** 1 Departments of Medicine and Pediatrics, Washington University School of Medicine, St. Louis, MO USA; 2 Department of Pediatrics, Washington University School of Medicine, Campus Box 8116, 660 S. Euclid Ave, St. Louis, MO 63110 USA; 3 Department of Pediatrics, Michigan Medicine, C.S. Mott Children’s Hospital, Ann Arbor, USA; 4 Department of Surgery, Washington University School of Medicine, St. Louis, MO USA

**Keywords:** HPV vaccine, Implementation strategies, CFIR

## Abstract

**Background:**

In the United States, the effective, safe huma papilloma virus (HPV) vaccine is underused and opportunities to prevent cancer continue to be missed. National guidelines recommend completing the 2–3 dose HPV vaccine series by age 13, well before exposure to the sexually transmitted virus. Accurate characterization of the facilitators and barriers to full implementation of HPV vaccine recommendations in the primary care setting could inform effective implementation strategies.

**Methods:**

We used the Consolidated Framework for Implementation Research (CFIR) to systematically investigate and characterize factors that influence HPV vaccine use in 10 primary care practices (16 providers) using a concurrent mixed methods design. The CFIR was used to guide collection and analysis of qualitative data collected through in-person semi-structured interviews with the primary care providers. We analyzed HPV vaccine use with data abstracted from medical charts. Constructs that most strongly influenced vaccine use were identified by integrating the qualitative and quantitative data.

**Results:**

Of the 72 CFIR constructs assessed, seven strongly distinguished and seven weakly distinguished between providers with higher versus lower HPV vaccine coverage. The majority of strongly distinguishing constructs were facilitators and were related to characteristics of the providers (knowledge and beliefs; self-efficacy; readiness for change), their perception of the intervention (relative advantage of vaccinating younger vs. older adolescents), and their process to deliver the vaccine (executing). Additional weakly distinguishing constructs that were facilitators were from outer setting (peer pressure; financial incentives), inner setting (networks and communications and readiness for implementation) and process (planning; engaging, and reflecting and evaluating). Two strongly distinguishing constructs were barriers to use, one from the intervention (adaptability of the age of initiation) and the other from outer setting (patient needs and resources).

**Conclusions:**

Using CFIR to systematically examine the use of this vaccine in independent primary care practices enabled us to identify facilitators and barriers at the provider, interpersonal and practice level that need to be addressed in future efforts to increase vaccine use in such settings. Our findings suggest that implementation strategies that target the provider and help them to address multi-level barriers to HPV vaccine use merit further investigation.

## Background

A vaccine targeting oncogenic human papilloma viruses (HPV) is an important health innovation. The HPV vaccine is predicted to prevent ~ 70% of cervical cancers as well as other genitourinary and oral cancers and genital warts in men and women. [1, 2] The vaccine has been recommended for routine use in the United States for over ten years [1] and already has been shown to prevent up to 99% of HPV-related cervical dysplasia, a precursor of cervical cancer. [1–3] However, the full benefit of the HPV vaccine can only be realized if the vaccination series is completed prior to exposure to the sexually transmitted virus. National guidelines recommend giving the vaccine to all adolescents at their 11 to 12 year old check-up visit concurrently with the tetanus, diphtheria, pertussis booster (Tdap) and the meningococcal conjugate vaccine (MCV4), and completing the HPV vaccine series before their 13th birthday. [4] Until a few months ago, the recommended series comprised 3 doses given over 6 months. At the end of 2016, this changed to 2 doses of the 9-valent vaccine given over 6 to 12 months, if the series is initiated before the 15th birthday. [5]

Introduced in the United States (U.S.) for girls in 2006 and for boys in 2011, use of this effective vaccine is far below the Healthy People 2020 objective of 80% coverage by age 13 to 15 for both sexes. [6] In 2015, only 41.9% of eligible females and 28.1% of eligible males aged 13–17 years had completed the 3-dose series, with marked variation among states. [7] Opportunities to prevent cancer continue to be missed. There is an obvious disparity in uptake of HPV vaccine when compared with coverage for Tdap (86.4%) and MCV4 (81.3%) in the same age-group. [7] Interventions to increase initiation of the HPV vaccine and series completion targeting both parents and providers that have been rigorously evaluated, have had at best a modest effect (~ 5% increase). [1, 8]^,^ [9] Many studies showing larger effects have been criticized for being methodologically deficient. [1, 9, 10] Alternative approaches to increase use of HPV vaccine are urgently needed. Accurate characterization of the facilitators and barriers to full implementation of HPV vaccine recommendations in the primary care setting could inform effective implementation strategies.

Closing the gap between the national goal and current vaccine use requires improved uptake in primary care settings where the majority of children are vaccinated. We hypothesized that in these settings, factors that influence HPV vaccine use are multifactorial and complex. The objective of this study was to use a systematic approach based on the Consolidated Framework for Implementation Research (CFIR) [11] to identify and characterize factors that are facilitators of and barriers to implementation of the national recommendations for HPV vaccine use. We will use this information to inform development of a pragmatic and effective implementation strategy to increase timely use of this important oncogenic vaccine.

## Methods

We assessed the implementation of HPV vaccine in ten community pediatric offices using a concurrent mixed methods design. Quantitative and qualitative data were analyzed separately and then were integrated to provide additional information. [12] Specifically, we conducted a retrospective analysis of HPV vaccine use data abstracted from medical charts and acquired during in-person semi-structured interviews with the pediatric primary care providers. The study was approved by the Washington University Human Research Protection Office.

The CFIR was used to guide development of the interview guide, data coding, and data analysis. Damschroder and colleagues developed the CFIR to consolidate and unify key constructs from 19 published implementation theories. [11] Seventy-two constructs are grouped under five major domains that influence successful implementation of evidence-based interventions. These include characteristics of the intervention (in this case, use of HPV vaccine in accordance with CDC guidelines), the inner setting (the pediatric practice), the outer setting (the context in which the pediatric practice resides), individual characteristics of the implementers (the healthcare providers), and the processes used for implementation of the intervention. [11] Definitions of the constructs within each domain can be found at 13012_2008_182_MOESM3_ESM.pdf [11].

### Study participants

We invited all members of the Washington University Pediatric and Adolescent Ambulatory Research Consortium (WU PAARC) to participate. WU PAARC is a practice-based research network of 79 community pediatricians and 6 pediatric nurse practitioners associated with Washington University. A convenience sample of 16 providers from ten practices volunteered to participate. Each participant provided written consent and received $50 as a token of appreciation for their time.

#### Advisory board

The study was guided by an Advisory Board of three pediatricians, one pediatric nurse practitioner, and two parents. The three pediatricians volunteered to be study subjects. The group met five times over the 12-month study period to review materials, problem solve, and assist in interpretation of study findings.

### Data collection

#### Quantitative data

For each provider, we abstracted data on vaccine coverage from medical charts for girls and boys who were 11 to 15 years old and had attended at least one office visit between January 1st, 2014 and December 31st, 2014. Data abstracted from the chart included: gender, date of birth, date of vaccine and type of vaccine (HPV, Tdap and MCV4).

#### Qualitative data

We developed an interview guide with a series of open-ended questions and probes to elicit examples that would clarify and illustrate the participant’s responses. The guide included general and specific questions that addressed the main CFIR constructs to determine those that supported or created barriers to implementation. We explored how each of these constructs differed from that used for other adolescent vaccines to gather information to explain the higher coverage for the other childhood vaccines. The interviews were conducted between 1/27/2016 and 5/24/2016, by the principal investigator (JG) and co-investigator (EW). Interviews were designed to last about 30 min and were conducted at the practices. All were digitally recorded and transcribed verbatim by trained transcriptionists. After the qualitative interview, participants completed a short, structured survey describing their practice arrangements.

### Data analysis

#### Quantitative data

For each practice and provider, we computed the overall coverage with HPV vaccine as the percentage of girls and boys who received three doses by their 13th birthday (a HEDIS measure for adolescent care). [13]

#### Qualitative data

We used a deductive approach to coding guided by the CFIR domains and constructs. We were also open to new themes that emerged inductively from the data. The transcripts were organized using NVivo software and each transcript was analyzed using consensual qualitative research methods with multiple analysts from diverse disciplines (e.g. pediatrician, implementation scientist, health services researcher) to foster multiple perspectives and validation. [14] We used an iterative process to develop a codebook that reflected each CFIR domain and its constructs, and key themes that emerged by refining the code definitions through consensus. The coding process began with two analysts who independently reviewed each transcript to identify the text units that were associated with each of the CFIR domains. Subsequently, they compared assessments, discussed differences and came to agreement on final text assignments. The text within each CFIR domain was further coded by three analysts to identify constructs within each CFIR domain. Thus, each transcript was subjected to a two-level consensus process.

#### Rating the CFIR constructs

Each transcript was subjected to a rating process. Five analysts used a deliberated consensus process to assign a rating to each construct within each transcript. The ratings reflected the valence (positive or negative influence) of the construct on implementation of HPV vaccine according to national guidelines (3 doses completed by the 13th birthday). Table 1 provides the criteria used to guide the rating process.

**Table 1:** Criteria Used to assign Ratings to Constructs

Rating	Criteria
-1	A negative influence on providing HPV vaccine according to guideline recommendations i.e., complete the 3-dose series by the child’s 13th birthday. The interviewee provides examples of how key aspects of the construct exert a negative impact on HPV vaccine use, reports an overall negative effect, or sufficient information is provided to make indirect inference about a negative effect.
O	Appears to have no effect on use of HPV vaccine according to guideline recommendations.
+ 1	A positive influence on providing HPV vaccine according to guideline recommendations i.e., complete the 3-dose series by the child’s 13th birthday. The interviewee provides examples of how key aspects of the construct exert a positive impact on HPV vaccine use, reports an overall positive effect, or sufficient information is provided to make indirect inference about a positive effect.

#### Integrating the rating of CFIR constructs and coverage rates

Next, for each provider, we integrated their construct ratings with their HPV vaccine coverage in a table (An example is provided in Table 2). We grouped physicians by coverage rates using tertiles: lower coverage < 5% (*n* = 5 providers), moderate coverage 6–19% (*n* = 6), and higher coverage > 20% (n = 5). This approach allowed us to observe patterns of ratings for CFIR domains and constructs among lower and higher coverage providers and generate hypotheses about constructs that distinguished between groups. We focused on extreme values of coverage to ascertain the most information. [14] Strongly and weakly distinguishing patterns were determined based on two dimensions within and across higher and lower coverage groups. We first assessed the pattern of positive and negative ratings for each construct within coverage groups (higher and lower) to determine if the factor was a facilitator or barrier to use of HPV vaccine. Next, we examined the difference in positive or negative ratings in the higher and lower coverage groups to determine if the construct was an important distinguishing factor. If this difference was ≥ 60% (e.g.,5/5 or 100% positive in higher coverage and 1/5 or 20% positive in lower coverage), the construct was considered to be “strongly distinguishing.” If this difference was between 40% to 59% (e.g., 5/5 or 100% positive in higher coverage and 3/5 or 60% positive in lower coverage), the construct was considered to be “weakly distinguishing.” If the difference was < 40% (e.g.,5/5 or 100% positive in higher coverage and 4/5 or 80% positive in lower coverage), the construct was determined to provide limited information about coverage rates. To support the validity of our conclusions, we checked with members of the Advisory Board. [14]

**Table 2:** An example of the table of integrated data from qualitative and quantitative studies comparing ratings for CFIR constructs in the characteristics of individuals domain and HPV vaccine coverage data (completion of 3 doses by 13th birthday)

CHARACTERISTICS OF INDIVIDUALS	1013	1004	1007	1015	1005	1001	1009	1020	1019	1008	1014	1018	1010	1006	1011	1003
HPV vaccine coverage	25.5%	24.6%	24.0%	23.0%	20.5%	18.1%	16.5%	16.1%	8.7%	8.1%	6.1%	4.8%	4.6%	4.2%	3.7%	1.2%
Knowledge and Beliefs																
Value completing the vaccine by age 13	+	+	+	+	+	+	+	–	+	+	+	–	–	–	+	–
Believes benefit in bundling	+	+	+	+	+	+	+	–	+	+	–	+	+	–	+	–
Self-Efficacy																
Enthusiastic use of HPV vaccine	+	+	+	+	+	+	+	+	+	+	+	+	+	–	+	–
Familiarity with facts about HPV vaccine	+	+	+	+	+	+	+	+	+	+	+	+	+	–	+	+
Confident to recommend strongly at early age (11–12)	+	+	+	+	+	+	+	+	+	+	+	–	–	–	+	–
Confident in ability to convince hesitant parents	+	–	+	+	+	–	+	+	+	+	–	–	–	–	+	–
Readiness to change	+	–	+	+	+	–	+	–	–	–	+	–	–	–	+	–

## Results

Sixteen providers participated in both the quantitative and qualitative studies. They comprised 15 pediatricians and one pediatric nurse practitioner. Five were male, 11 were female, 13 were Caucasian, 2 were African American, and one was Asian. The 16 providers were drawn from ten practices, nine of which had multiple providers (2–6 providers per practice). Two practices were part of a healthcare group and had access to central support for information systems and quality improvement staff. Such resources were not available at other practices. Eight practices used an electronic medical record (EMR).

### Quantitative data

Medical records from a total of 4592 teens were eligible for inclusion in the data analysis of coverage rates. Overall, 13.9% of eligible teens had received 3-doses of HPV vaccine before their 13th birthday (16.9% girls, 11.3% boys). This metric varied among practices from 2.3% to 25.5% and there was wide variability in HPV vaccine coverage among providers within the same practice (Table 3). Consequently, we chose to analyze the qualitative data at the level of the individual provider rather than at the level of the practice.

**Table 3:** Coverage Rates for HPV Vaccine by Practice and Provider

Practice	Number of children age 13	Electronic medical record (EMR) or paper records	Completed 3 doses HPV by 13th birthday	
			Practice coverage	Provider coverage
A	329	EMR	25.5%	25.5%
B	383	EMR	23.0%	23.0%
C^a^	299	EMR	18.1%	18.1%
D	726	EMR	18.6%	24.0%, 3.7%
E	441	Paper records	13.8%	24.6%, 16.1%, 4.2%
F^a^	940	EMR	10.6%	20.5%, 4.6%
G	636	EMR	9.6%	16.5%, 8.1%
H	416	EMR	8.7%	8.7%
I	293	EMR	6.1%	6.1%
J	129	Paper records	2.3%	4.8%, 1.2%
All	4592		13.9%	

^a^Practice was part of a healthcare group with access to information system assistance and quality improvement staff

### Qualitative data

CFIR constructs are not reported if we found limited text that could be coded within that domain construct. For intervention domain, this included the following constructs: intervention source, evidence strength and quality, trialability, design quality and packaging. For the inner setting domain, this included: structural characteristics, culture, compatibility, organizational incentives and rewards, and learning climate. For the characteristics of individuals domain, we found no text about individual identification with the organization. Within the process domain, we did not identify any opinion leaders, internal implementation leaders or champions.

### Innovation characteristics domain

#### Relative advantage

The most commonly mentioned perceived advantages to providing HPV vaccine at age 11–12 years rather than to older teens were increased immunogenicity, successfully completing the series before exposure to HPV and accessing the child at their 11–12 year old check-up required by school. Although the vaccine can be given to children as young as 9, no providers did so routinely. Several providers offered it to younger children accompanying an older sibling to a check-up visit where they received HPV vaccine.

#### Adaptability

All providers adapted the vaccine dosing schedule, most often to provide the 2nd and 3rd doses at subsequent annual checkup visits. Some providers also delayed the age of initiation beyond 11–12 years to prevent teens from having three vaccines concurrently or to avoid conflict with a family they thought might be uncomfortable discussing prevention of a sexually transmitted disease at this age.

#### Complexity

The majority of providers identified at least one difficulty with administering the 3-dose series. Many cited the resistance and hesitancy of parents, as exemplified by this quote:

“Well, I think you have to go in more proactively with that one [HPV], because you expect a fight. You do, because nine times out of ten, you have a parent who has heard something about it who has a problem with it. You are trying to push the vaccine in a positive way before the negative parts come in from the parent.”

Others mentioned that it took longer to discuss HPV vaccine than other adolescent vaccines and less frequent visits of older adolescents increased the difficulty of completing the series.

#### Cost

Few providers mentioned cost associated with providing HPV vaccine, although several noted that providing the 3-dose series was revenue generating.

### Outer setting domain

#### Patient needs and resources

Most providers felt it was important to provide information about vaccine benefits as reported by this provider:

“I think there are a lot of negative messages out there, so it’s important for the families to hear the positive message from us.”

To address this need, they routinely informed parents that HPV vaccine prevents cancer and sometimes they mentioned the prevention of warts. However, few routinely discussed vaccine safety, preferring to provide this information if parents expressed specific safety concerns. Few used parent educational materials apart from the required vaccine information sheets (VIS) from the CDC. Several providers noted that teenagers often were concerned about getting the HPV vaccine as they had heard it was more painful than other vaccines, and some teens were reluctant to get all three adolescent vaccines at the same visit.

#### Peer pressure

A few providers described pressure from a partner in their practice to change their approach to HPV vaccine implementation.

#### External policy and incentives

All providers were aware of the national guideline recommendations to complete the 3-dose series by age 13. Many parents chose to defer HPV vaccine as it is not required for school attendance. Few providers were aware of financial incentives including those offered by vaccine manufacturers to increase completion of the 3-dose series and by Medicaid to promote meaningful use of clinical information.

#### Cosmopolitan

All providers reported that they learned about the recommendations for HPV vaccine from external sources, most commonly through membership with the American Academy of Pediatrics (AAP) list serve. They also received information from “throw away” medical journals, drug representatives, and the internet, and were unlikely to seek or receive information about the vaccine from local infectious disease experts.

### Inner setting domain

#### Networks and communication

Except for the solo-practitioner, all providers reported formal or informal opportunities for communication with other providers in their practice, typically through formal meetings or shared office space. However, the majority of providers were unaware of how their colleagues approached delivery of HPV vaccine.

#### Readiness for implementation

Few providers reported leadership engagement in system-level improvement activities or use of available resources (such as their EMR and reminder calls) to increase use of HPV vaccine. Only one provider reported training for office staff involved with vaccine delivery.

#### Implementation climate

In some practices that delivered both the other adolescent vaccines at the 11–12 year old check-up visit, delivery of HPV vaccine fit well with existing workflows. In others, delivery required additional work at a subsequent check-up visit. More than half of providers reported that increasing use of HPV vaccine was a relatively high priority for improvement within their practice, but there were no statements that demonstrated a tension for change. Although all aspired to 100% vaccine coverage, none had any formal goals for themselves or their practice, or monitored vaccine coverage.

### Characteristics of individuals domain

#### Knowledge and beliefs

All providers believed that HPV vaccine was an important vaccine and had a personal investment in providing the vaccine. Several routinely provided vaccine-related facts about HPV vaccine to parents such as usage data to address safety concerns. The majority of providers were aware that earlier vaccination resulted in an increased immune response. They varied in their belief that the vaccine should be initiated at age 11–12 years, and that there was benefit to initiating the HPV series by bundling with Tdap and MCV4. While some were very supportive of this “bundling,” others thought it was “a harder sell.” Several providers were reluctant to immunize pre-pubertal teens and tailored their recommendation to their perception of the child’s risk of sexual activity. One provider mentioned concern that the duration of protection may not be adequate if provided at age 11–12.

#### Self-efficacy

Providers varied in their confidence to strongly recommend HPV vaccine at the 11–12 year visit and to convince hesitant parents to accept it. Many expressed frustration with hesitant parents as demonstrated by this quote:

“You can talk until you’re blue in the face and give every ounce of evidence and research behind what you do and why you do it. They don’t listen. It just—it’s frustrating as a provider. Extremely frustrating.”

#### Readiness to change

Several providers had engaged in informal efforts to change their approach to increase vaccine use. An example is described by this provider.

“I used to say, “I have an optional vaccine.” And, I found that when I said that, I had a very, very low, um, percentage of patients who agreed to take it. So, I don’t use the word ‘optional’ anymore.”

### Process domain

#### Planning

No-one used a formal planning process initially to implement HPV vaccine or subsequently to change their approach to vaccine use.

#### Engaging

Few providers had actively engaged staff members in problem solving to increase vaccine use or expected their Medical Assistants (MAs) to endorse vaccination.

“[The MA’s] don’t really influence the parents’ decisions about vaccines…They administer the vaccines, but they’re not counseling.”

#### Executing

Providers universally assumed responsibility for vaccine delivery and reported the same approach for girls and boys. Typically, they recommended the HPV vaccine at the 11–12 year old visit, although the strength of this recommendation varied. Some providers routinely shared personal information about vaccinating their children or grandchildren, while others only provided this information if asked. One provider had the MA present all three vaccines as “the vaccines recommended today,” and only discussed benefits and concerns if requested by the parent. He suggested that this MA-driven process of presenting this vaccine as “no different from any other vaccine” was very effective. He thought that the more traditional approach of “We’re gonna do this vaccine today, this vaccine today, and your doctor will discuss this vaccine with you today. …” both “justified the concern that families have if they came in with it and it created concern in families that didn’t.”

Teamwork to deliver the vaccine series was uncommon. Many providers were unaware of the approach their MA took with parents regarding follow-up doses after they had immunized them with the first dose.

All providers reported that they had vaccine-only visits and offered follow-up doses at acute care visits. Few used practice-level strategies to support subsequent doses such as routinely booking follow-up appointments or making reminder calls to parents. Most often the responsibility to schedule the 2nd and 3rd doses was left to the parent.

#### Reflecting and evaluating

No providers reviewed HPV vaccine coverage data. One provider commented that, “it’s not as easy as you think to get the data out of here [the EMR].”

### Integrated data results

Of the 72 CFIR constructs assessed in the table with the coverage rates, seven strongly distinguished and seven weakly distinguished between providers with higher versus lower HPV vaccine coverage. These constructs are briefly described here and are summarized in Table 4 as facilitators and barriers to vaccine use.

**Table 4:** Facilitators and Barriers for completing the HPV vaccine series by 13th birthday

CFIR Domain	Facilitators	Barriers
Characteristics of HPV vaccine recommendations	*Perceived advantage to vaccinate at age 11/12 compared to older: • Increased immunogenicity • Efficient; Can bundle • Complete series before exposure • Easier patient access	*Adapt recommendation to initiate at older age
External environment and context	Financial incentives for series completion: • Increased billing • Meaningful use for Medicaid	*Not mandated by school therefore offer as optional at age 11/12
Internal context of practice	• Communication across staff and providers to coordinate series completion	
	• Leadership engaged in system level improvements e.g., EMR	
	• Use of multiple available resources e.g., EMR alert, outreach calls	
Characteristics and attitudes of providers	• *Perceive value in completing series by age 13	
	• Perceive value in bundling 3 adolescent vaccines	
	• *Confident to strongly recommend HPV vaccine and to convince hesitant parents	
	• Enthusiastic about HPV vaccine	
	• *Already made efforts to increase HPV vaccine use	
Process for implementation of HPV vaccine recommendations	• *Routinely provide strong recommendation for HPV vaccine at age 11/12 and bundle 3 vaccines	
	• Involve staff in meaningful problem-solving	
	• Reflect on HPV vaccine use with a view to making changes	

*Strongly distinguishing factor

### Intervention characteristics domain

Within the intervention characteristics domain, relative advantage and adaptability of the age of initiation were strongly distinguishing constructs, one acting as a facilitator and one as a barrier to vaccine use. All providers in the higher coverage group had positive ratings for perceiving a relative advantage to providing the vaccine at age 11–12 years as suggested by the guidelines compared to delaying until the teen is older, as characterized in this quote.

“We would recommend the Tdap, the Menactra, as well as the HPV. …We recommend starting the HPV at that age, simply because the earlier you start it, the more effective it becomes. You want to start it because you want to get the kids before there’s any concern for any exposure. You wanna make sure that they complete their series. We always routinely recommend that at 11.”

None of the providers with higher coverage routinely adapted the recommended age of vaccine initiation. Providers with lower coverage routinely initiated the vaccine series at a later age when they described the parents as “more accepting.”

### Outer setting domain

Within the outer setting domain, patient needs and resources was a strongly distinguishing construct. Providers who anticipated that parents wanted to know which vaccines offered at the 11–12 year old checkup were mandatory for school attendance routinely suggested HPV vaccine as optional at that visit. These providers also had lower vaccine coverage suggesting this construct is a barrier to HPV vaccine use. One provider described her typical approach:

“Because the Menactra is, and the Tdap are required by school, I guess I introduce them by saying these are the vaccines they get at 11, in order to go to school. The other one I say we highly encourage that your child gets the HPV.”

None of the providers with higher coverage adjusted the adolescent vaccine recommendation in this way and they were more likely to routinely discuss vaccine safety than those with lower coverage. Other factors in this domain weakly distinguished between higher and lower vaccine coverage. Providers with higher coverage were less likely to feel peer pressure to change their implementation approach and were more likely to have positive ratings for responding to financial incentives as demonstrated by this quote:

“…… we're imagining that a lot of the parameters they're using for meaningful-use will also be for pay for performance… and so we wanna make sure our percentages are up there so, we're, we're always very cognizant of those red flags (… *to indicate a vaccine is due)* and, and that they need to be checked off.” … “That, that's the main incentive. Um, you know. Meaningful use.”

These findings suggest that a statement of vaccine safety and awareness of financial incentives can facilitate vaccine use.

### Inner setting domain

Within the inner setting domain, patterns of ratings in the dimensions of networks and communications and readiness for implementation weakly distinguished provider-level vaccine coverage, acting to facilitate use. Providers who communicated with staff to coordinate vaccine use; reported a consistent approach across providers in their practice; described leadership engagement in system-level improvement activities and use of available resources to provide HPV vaccine had higher vaccine coverage than those who did not report these features.

### Characteristics of individuals domain

Within the characteristics of the individual domain, many factors distinguished between higher and lower vaccine coverage, all facilitating vaccine use. The knowledge and beliefs of the providers about HPV vaccine was a strongly distinguishing construct. Providers with higher coverage were more likely to believe there was value in completing the vaccine series by age 13 and in bundling the three recommended adolescent vaccines at the 11–12 year checkup visit. Provider self-efficacy was a strongly distinguishing construct as was readiness to change of individual providers. Providers with higher coverage were more likely to be enthusiastic and confident in strongly recommending the vaccine at age 11–12 and in their ability to convince hesitant parents to accept the vaccine as demonstrated by this quote:

“…I feel like I’m able to work through their concerns, rebut any inaccuracies that they come in with, and give them the right information they need to make a decision to proceed.”

In contrast, those with lower coverage did not share this confidence as demonstrated by this quote:

“…typically, if they're not going to give it, nothing you’re going to tell them is going to change their mind.”

Those with higher coverage were more likely to already have engaged in personal efforts to increase HPV vaccine use such as adding provider reminders to the EMR.

### Process domain

Within the process domain, executing was a strongly distinguishing construct. All providers with higher coverage routinely strongly recommended and routinely provided the vaccine at the 11–12 year old check-up visit, and bundled the three adolescent vaccines. Also, they were more likely to provide personal information about HPV vaccine use as described by one provider:

“I give a quick spiel about how I think it’s really important, how it helps protect against cancer, how it’s safe, we’ve given lots of doses, nationwide lots of doses have been given, it’s effective, it prevents cancer, I give it to my kids and lots of kids every day, I think it’s really important…”

Several constructs weakly distinguished between providers with higher and lower coverage. Evidence of planning, engaging, and reflecting and evaluating was most often associated with higher coverage, suggesting these constructs facilitate use.

## Discussion

Study findings both confirm that effective strategies to increase HPV vaccine use are urgently needed and inform their development. By systematically examining the complex web of factors that influence use of this vaccine in the primary care setting, we identified important facilitators and barriers to following National guidelines. We learned that a multi-level approach is needed to markedly increase use of this important vaccine. At the provider-level, buy-in to vaccination by age 13 is critical as is the provider’s confidence to address parental hesitancy. At the interpersonal-level, our findings suggest several strategies the provider could use with the patient, parents and staff to increase vaccine use. These include recommending the three adolescent vaccines as a bundle and addressing parental hesitation with a strong and persistent recommendation for timely HPV vaccine use. Better co-ordination between the provider and staff who administer the vaccine could standardize communication messages for patients and parents and ensure opportunities for vaccination are not missed. At the practice-level, collaboration among providers could improve efficiency by standardizing use of reminder systems and maximize benefit from financial incentives.

Our findings confirm that the provider’s approach to HPV vaccine personally, interpersonally and at the practice level, strongly influences uptake. [15, 16] Providers in these independent community practices universally assumed responsibility for vaccine delivery, and reported they routinely recommended HPV vaccine, Tdap and MCV4 to their patients at their 11–12 year-old checkup visit. Our analysis demonstrated that the quality of their recommendation varied widely as did HPV vaccine coverage by age 13. These findings are consistent with national studies with larger samples [17, 18] and likely reflect the complexity of communication about this vaccine. [18] Failure to require HPV vaccine for school attendance influenced some providers to suggest that the vaccine could be delayed [19] and anticipation of parental resistance caused them to feel compelled to have a conversation about the vaccine. This likely contributed to reduced vaccine uptake as parents are more likely to resist vaccination recommendations if the provider uses a participatory, conversational approach rather than a more directive approach (a presumptive or announcement approach). [20, 21] It is likely that providers’ lack of buy-in to vaccination by age 13 and their low self-efficacy to address parental hesitancy further compromised their ability to provide a strong recommendation. At the practice-level, reminder and recall systems that can increase vaccine series initiation and completion, [9, 10, 22, 23] were infrequently used. Many providers in our sample lacked resources such as information technology (IT) support to augment their EMR with these systems. [24]

Effective implementation strategies must help providers to change their approach to HPV vaccine. However, changing provider behavior is difficult. Educational interventions targeting providers have not been successful, [9] and reminders and audit and feedback have had mixed results. [9] Although lack of a strong provider recommendation is reputed to be the most common barrier to use of HPV vaccine, [15–17, 25] few studies have tried to improve the quality of provider communication. One randomized trial conducted in the primary care setting showed that training providers to use an announcement approach increased vaccine coverage by 5%, [8] but the broader literature of message framing has yielded mixed results. [18] In addition to changing provider behavior regarding the timing and quality of their recommendation for HPV vaccine, our findings suggest the need for providers to develop an adolescent vaccine delivery system. While a systematic approach was effective in a large healthcare system with access to staff and technical support for implementation, [19] most interventions to systematize vaccination in primary care practices have been disappointing and expensive. [9] [26] A recent randomized controlled trial showed an intervention comprised of provider education, audit and feedback and EMR reminders increased vaccine initiation by 8% and the intervention was relatively inexpensive, but may not be scalable without IT support. [27] Effective, scalable interventions to encourage and support providers in independent practices to deliver HPV vaccine to all eligible teens in a timely fashion are needed. [10]

Our findings inform an implementation strategy to change provider behavior regarding this complex vaccine. An acceptable approach will be simple to use, save time, and alleviate the stress associated with dealing with vaccine hesitant parents. [28] Key ingredients might include the following: 1) Education to motivate behavior change and increase self-efficacy. Providers must be convinced of the benefits of early initiation of the vaccine series and able to succinctly and comfortably address parental concerns. 2) To facilitate change, additional pragmatic resources such as a communication strategy to increase the quality of vaccine recommendation are needed. 3) Targeting increased coverage for any age before the 15th birthday may engage more providers in the change process and capitalize on the latest recommendation for a 2-shot series. [5] It would also significantly impact the Healthy People 2020 goal of 80% coverage for 13–15 year olds. 4) Finally, engaging staff in developing and implementing a new vaccine delivery system to increase vaccine use would be beneficial. Failure to simultaneously address all these factors may explain why prior interventions to improve use of HPV vaccine have had at best a modest effect and changes have not been sustained.

Our study is limited by several considerations. Although we believe the study sample is representative of providers working in independent, community-based pediatric practices in our community with considerable variation in the context of where and how the providers practiced, our convenience sample of 16 providers is small and from one geographic area and may not be representative of other populations. Completion of the 3-dose series before the 13th birthday was low among participants, consistent with national data. [7] As no providers had high vaccine uptake, our findings regarding barriers and facilitators for HPV vaccine uptake should be considered as exploratory. The time span for collecting the quantitative and qualitative data were different (2014 and 2016 respectively. National data show the increase in HPV vaccine coverage from 2014 to 2015 was minimal, and suggest that a dramatic change between 2014 and 2016 is unlikely. [7, 29] However, if changes in coverage were large and not equal across providers, then our assessment of barriers and facilitators may be inaccurate. Finally, our qualitative data was retrospective and we did not interview parents or patients.

## Conclusions

In conclusion, study and national data suggest that many teens are not protected against HPV-related cancers prior to exposure to the virus and that widespread efforts to increase HPV vaccine use are urgently needed. Using CFIR to systematically examine the use of this vaccine in independent primary care practices enabled us to identify facilitators and barriers at the provider, interpersonal and practice level that need to be addressed in future efforts to increase vaccine use in such settings. Our findings suggest that implementation strategies that target the provider and help them to address multi-level barriers to HPV vaccine use merit further investigation.

## Abbreviations

AAP: American Academy of Pediatrics
ACIP: Advisory Committee on Immunization Practices
CDC: Centers for Disease Control and Prevention
EMR: Electronic Medical Record
HEDIS: Healthcare Effectiveness Data and Information Set
HPV: Human Papillomavirus
IQR: Inter-quartile range
MCV4: Quadrivalent meningococcal vaccine
NCATS: National Center for Advancing Translational Sciences
NIH: National Institutes of Health
Tdap: Tetanus, diphtheria and pertussis vaccine
U.S.: United States
VIS: Vaccine information sheet
WU PAARC: Washington University Pediatric and Ambulatory Research Consortium
